# Recent Advances in Diagnosis, Prevention, and Treatment of Human Respiratory Syncytial Virus

**DOI:** 10.1155/2013/595768

**Published:** 2013-12-09

**Authors:** Swapnil Subhash Bawage, Pooja Munnilal Tiwari, Shreekumar Pillai, Vida Dennis, Shree Ram Singh

**Affiliations:** Center for NanoBiotechnology Research, Alabama State University, Montgomery, AL 36104, USA

## Abstract

Human respiratory syncytial virus (RSV) is a common cause of respiratory infection in infants and the elderly, leading to significant morbidity and mortality. The interdisciplinary fields, especially biotechnology and nanotechnology, have facilitated the development of modern detection systems for RSV. Many anti-RSV compounds like fusion inhibitors and RNAi molecules have been successful in laboratory and clinical trials. But, currently, there are no effective drugs for RSV infection even after decades of research. Effective diagnosis can result in effective treatment, but the progress in both of these facets must be concurrent. The development in prevention and treatment measures for RSV is at appreciable pace, but the implementation into clinical practice still seems a challenge. This review attempts to present the promising diverse research approaches and advancements in the area of diagnosis, prevention, and treatment that contribute to RSV management.

## 1. Introduction

Worldwide, there are reportedly about 12 million severe and 3 million very severe cases of lower respiratory tract infection (LRTI) in children [1]. Respiratory syncytial virus (RSV) is a common contributor of respiratory infections causing bronchiolitis, pneumonia, and chronic obstructive pulmonary infections in people of all ages but affects mainly children and elderly along with other viral infections leading to high mortality and morbidity [2–4]. A recent global survey suggests that RSV is not prevalent throughout the year in the tropical regions of the globe, but the incidence peaks in winter with a wide ranging persistence depending on the geographical topology [5]. RSV has been reported to be a prevalent lower respiratory tract pathogen distributed worldwide including countries from both, the developed and developing world. The major countries with RSV seasonal outbreaks include USA, Canada, Cambodia, Mexico, Uruguay, Brazil, Peru, France, Finland, Norway, Sweden, Latvia, Denmark, Germany, Netherlands, Ireland, Italy, Turkey, Iran, Saudi Arabia, Australia, New Zealand, China, Korea, Hong Kong, Japan, India, Pakistan, Bangladesh, Nepal, Taiwan, Vietnam, Myanmar, Thailand, Madagascar, Kenya, Zambia, Nigeria, and Columbia. The data about human RSV described in literature over the years seem to have been unchanged significantly, indicating the severity of RSV and the urgent concern to address this issue. An estimate of more than 2.4 billion US dollars per year is the economic cost of viral lower respiratory tract infection in children [6].

**Figure 1:**
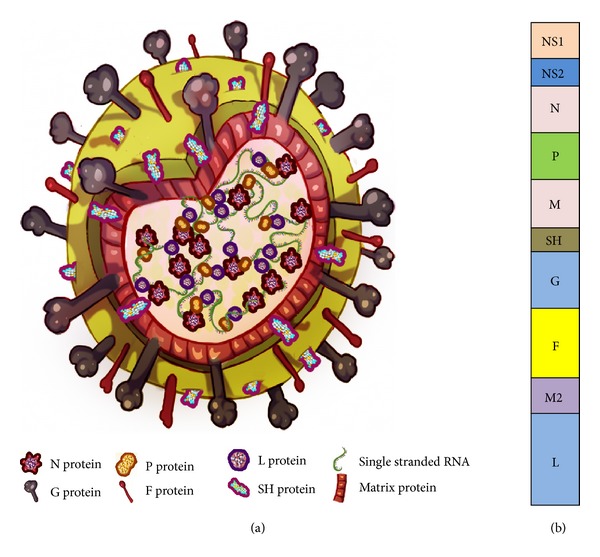
Structure and genome organization of respiratory syncytial virus. (a) Approximately 200 nm RSV virion particle and (b) single stranded negative RNA genome consisting of 10 genes.

RSV is a *Paramyxovirus* belonging to the genus *Pneumovirus*. RSV is an enveloped, nonsegmented, negative, single stranded linear RNA genome virus (Figure 1). RSV genome (~15 kb) has 10 genes encoding 11 proteins with two open reading frames of gene M2 [7, 8]. Other genes include nonstructural proteins NS1 and NS2 (type I interferon inhibitors), L (RNA polymerase), N (nucleoprotein), P (Phosphoprotein cofactor for L), M (Matrix protein), M2.1 and M2.2 (required for transcription) SH (small hydrophobic protein) G (glycoprotein), and F (fusion protein). Being a negative strand RNA genome virus, RSV packages its own polymerase into the nucleocapsid. Of these proteins, fusion protein (F) is indispensable for viral attachment to the host and entry into the host cell. Although the G protein is responsible for the preliminary attachment, the F protein is necessary for the fusion, budding, and spread of the virus [9, 10]. After attachment to the host cell, RSV fuses with the host cell membrane using the F protein through the 6 helix coiled-coil bundle of the F protein, a mechanism characteristically found in *paramyxoviridae* members [11]. Although the detailed mechanism of RSV infection is not fully understood, the most accepted mechanism is the entry of the nucleocapsid into the host cell mediated by the F protein through clathrin mediated endocytosis [12]. The RNA is first converted into a plus strand, which serves as the template for replication; whereas for transcription, the RNA genome itself transcribes mRNA for protein synthesis without any intermediate.

**Figure 2:**
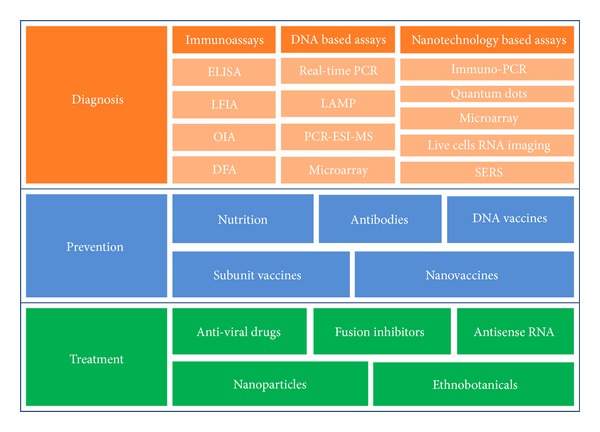
A schematic representation of RSV management through coordinated diagnosis, prevention, and treatment.

Almost all children of 2 years of age will have had an RSV infection and leading to 160,000–600,000 deaths per year [4]. Approximately, 25% to 40% of infants and children at the first exposure to RSV have signs or symptoms of bronchiolitis or pneumonia. These symptoms include rhinorrhea, low-grade fever, cough, and wheezing. The symptoms in adults may include common cold, with rhinorrhea, sore throat, cough, malaise, headache, and fever. It can also lead to exacerbated symptoms such as severe pneumonia in the elderly, especially residing in nursing homes [13]. Usually, children show symptoms within 4 to 6 days of infection and most of them recover in 1 to 2 weeks while serving as carriers of the virus for 1 to 3 weeks. RSV infection in children of nosocomial origin is associated with higher mortality than community-acquired illness because of the pre-existing morbidity [14, 15]. Severe RSV disease risk hovers for the elderly and adults with chronic heart or lung disease or with weakened immune system [16]. RSV infection does not provoke lasting immunity [17] therefore, reinfection is very common [18]. Recently, RSV infection was reported to account for hospitalizations and mortality in elderly people [19]. RSV accounted for severe lower respiratory tract infections including chronic lung disease, systemic comorbidities, and even death. At present, there is no specific treatment for RSV infection ever since its first discovery in 1956 [20]. Currently, Food and Drug Administration (FDA) approved prophylactic drug for RSV that includes palivizumab and ribavirin; administered along with symptomatic treatment drugs and supportive care. Currently, techniques used for diagnosis of RSV include ELISA, direct immunofluorescence, western blot, PCR, and real-time PCR. The diagnosis and treatment scenario has significantly changed with the advent of advanced techniques and in-depth understanding of RSV biology, but the execution of these clinical developments in practice requires extensive study and time. This review presents the recent advances in the diagnosis, prevention, and treatment of RSV (Figure 2).

## 2. Diagnosis

There are ten known genotypes of RSV based on the sequence analysis of the RSV genome, which suggests that the pathogen changes with time and the resulting genotypes pose a threat to the public health. Information from comparative genome analysis has been utilized for evolutionary studies and most importantly for the development of detection and treatment studies [21, 22]. Diagnostic assays for detection of pathogens have a pivotal role in public-health monitoring [23]. However, diagnosis becomes difficult when the causative agent exhibits overlapping symptoms with other disease(s) or remains nonsymptomatic. Hence the correct diagnosis has to be empirically derived for the treatment. Some of the promising molecular and biophysical techniques for RSV diagnosis are discussed below (Table 1).

### 2.1. Immunoassays

#### 2.1.1. Enzyme-Linked Immunosorbent Assay

Enzyme-linked immunosorbent assay (ELISA) is the enzyme facilitated colorimetric detection of specific protein-antibody complexes. ELISA is extensively used for detection of various proteins at very low concentration in different sample types, thus making it clinically significant in routine diagnosis of pathogens. ELISA for RSV detection is mainly based on targeting RSV F protein (antigen). Recently, several modifications of the classical ELISA technique have been efficiently developed and employed for the detection of RSV. Sensitivity of ELISA was increased by using the high affinity anti-RSV F antibody peptides derived from the motavizumab [85]. Motavizumab is a high affinity antibody based therapeutic against RSV which binds to RSV F protein; however, it was disapproved by the FDA due to higher hypersensitivity in patients receiving motavizumab as compared to palivizumab. Motavizumab also caused urticaria [34]. It is presumed to be a better binding target than the conventional F protein ELISA. This method could prove more effective than the F protein ELISA due to its higher sensitivity to the degradation of motavizumab. In a simple thin layer amperometric enzyme immunoassay, RSV was detected as early as 25 minutes, at low cost with comparative sensitivity to that of real-time PCR and immunofluorescence assays [35]. The assay is based on the development of a double layer sandwich method similar to ELISA. It involves a polystyrene microarray slide coated with monoclonal antibody which captures the antigen (RSV). The antigen-antibody complex is detected by horse radish peroxidase conjugated secondary antibody on a screen printed electrochemical cell coated with the substrate.

#### 2.1.2. Immunofluorescence Assay

Presently, immunofluorescence is one of the most common and rapid RSV detection techniques used, where the antigen is detected by a fluorescently tagged antibody. Direct fluorescent antibody assay (DFA) is a standard detection technique used for decades and other RSV detection techniques are often compared to DFA for evaluating their efficiency [24]. An indirect assay which uses secondary fluorescently tagged antibody is another option to DFA. Currently, there are many molecular techniques that are more sensitive and reliable than DFA, but DFA is widely used for RSV detection in clinical samples due to the ease and rapidity. The sensitivity and specificity of DFA are a subject of variance as the success of the technique is dependent on numerous factors, mainly the skills of the technician and nature of the sample. A study showed that DFA could detect RSV with a sensitivity and specificity of 77.8% and 99.6%, respectively (positive predictive value was 98.6% and the negative predictive value was 94%). DFA is a reliable technique for RSV detection for patients tested within first 3 days after onset of symptoms, but the sensitivity decreases if tested 4–7 days after the onset of the symptoms [24]. DFA can detect RSV alone or as a multivalent test for other respiratory viruses (influenza A and B viruses, parainfluenza virus types 1 to 3, and adenovirus) using SimulFluor Respiratory Screen assay [25].

#### 2.1.3. Optical Immunoassay

The direct visualization of an antigen-antibody complex often referred to as optical immunoassay (OIA) is used for qualitative detection of *Streptococcus* and influenza virus. Visualization of immunocomplexes can be enhanced by immobilizing them on a special reflecting surface. The antigen-antibody complex forms a thin layer, which changes the reflective properties of the surface [36]. The technique is simple, rapid, and sensitive and as low as 1 ng of antibody per mL can also be detected [37]. OIA is now used for detection of RSV with a sensitivity, specificity, positive, and negative predictive values of 87.9%, 99.6%, 98.9%, and 94.5%, respectively. Though OIA offers rapid and cost-effective RSV detection, it is recommended that negative results of OIA should be confirmed by other tests [38].

#### 2.1.4. Lateral Flow Immunoassay

Lateral flow immunoassay (LFIA) is an immunochromatographic technique known for the rapid detection of RSV from nasal washes or nasal aspirates. The lateral flow of antigen-antibody complex on the substrate matrix reaches the reaction area and results in formation of colored band indicating the presence of antigen in the specimen. There are many LFIA kits like Remel Xpect, Binax Now RSV, BD Directigen EZ RSV, QuickLab RSV Test, and RSV Respi-Strip [39–42]. The sensitivity and specificity are normally above 90% and 95%, respectively, but vary as per the manufacturer. Modifications of LFIA may include colloidal gold conjugated with antibody specific to RSV in the matrix for trapping the antigen and assisting the gold mediated reaction for color band development.

### 2.2. Loop-Mediated Isothermal Amplification

Loop-mediated isothermal amplification (LAMP) is a nucleic acid based detection method used for bacterial and viral pathogens. It can be used for RNA viruses where the additional step of cDNA synthesis is required and commonly designated as reverse transcription LAMP (RT-LAMP). LAMP consists of pairs of primers specific to cDNA, which is then amplified by autocycling strand displacement activity of DNA polymerase generally at 60°C. The reaction time of 1 to 1.5 hours is sufficient and can be monitored by real-time turbidimeter and the resulting product can also be viewed by agarose gel electrophoresis. This method can distinguish RSV strain A and B upon restriction digestion of the product [41]. Alternatively, RSV strain A and B can be detected designing specific primer sets for them. The efficiency of RT-LAMP was tested for RSV detection from nasopharyngeal aspirates and compared with viral isolation, enzyme immunoassay, immuno-chromatographic assay, and real-time PCR. RT-LAMP was the most sensitive among all the methods tested, with the exception of real-time PCR. Also, the RT-LAMP was specific for RSV and did not react with any other respiratory pathogens like RNA viruses, DNA virus, or bacteria [43]. Although this method is rapid, the requirement of the turbidimeter makes it slightly inconvenient as a cost-effective detection technique. Development in the chemistry of the LAMP has made it possible to get rid of the turbidimeter as it is now possible to visually detect the presence of pathogen in the test sample. LAMP is now referred to as colorimetric detection of loop-mediated isothermal amplification reaction due to incorporation of the dye in the chemistry to aid visual detection. Wang et al. (2012) have developed such an RT-LAMP assay for the detection of human *metapneumoviruses* (which include rhinovirus, RSV, influenza virus A/PR/8/34 (H1N1)), which can detect as low as ten viral RNA copies with better efficiency than RT-PCR [44]. It is now possible to detect RSV A and B strains in nasopharyngeal specimen using multiplex LAMP (M-LAMP) in just 30 minutes. M-LAMP had a sensitivity and specificity of 100% when compared with PCR [45].

### 2.3. Polymerase Chain Reaction (PCR) Based Detection

#### 2.3.1. Conventional PCR

The applicability of PCR is ubiquitous and has made the diagnosis of pathogens tremendously rapid and sensitive [46]. RSV is usually challenging to detect due to poor viral titer and sensitivity to antigen based detection methods. Thus, a PCR based method was developed and compared to serological and culture based detection methods in adults having respiratory infections [47]. The PCR method was based on the reverse transcription-nested PCR technique involving the outer and inner primers designed from the F gene of RSV strain A, over a two day procedure with a sensitivity of 73%. This method is faster as compared to the conventional culture method which usually takes 3–5 days, resulting in faster treatments.

#### 2.3.2. Real-Time PCR

Although conventional reverse transcriptase-PCR (RT-PCR) is sensitive as compared to the culture methods, it suffers from the lower sensitivity. This problem has been obviated by the development of real-time PCR based methods. Real-time PCR is by far the most sensitive method for the detection and diagnosis of a wide array of pathogens, including RSV. Several studies have been conducted for the development of real-time PCR assays for RSV detection, especially the RT-PCR. In one such study, a rapid, sensitive, and specific method was devised based on TaqMan real-time PCR for the detection of both RSV A and RSV B strain [48]. The primer and the probe sets were designed from the nucleocapsid gene (N). The sensitivity of this method was found to be 0.023 PFU (plaque forming unit) or two copies of mRNA for RSV A, whereas the sensitivity for RSV B was 0.018 PFU or nine mRNA copies. This method is fast and efficient as the diagnosis was performed within 6 hours of the procurement of samples.

A similar assay based on real-time PCR was developed and employed in the detection of RSV in the bronchoalveolar lavage (BAL) of lung transplant patients or patients with respiratory infections [49]. The assay was designed based on the RSV N gene and first involved a screening step of the RSV positive samples using a SYBR green based assay, followed by quantitative real-time PCR using TaqMan based assay to be more cost effective. Also, the assay was developed for the detection of both RSV A and B subgroups separately. The assay was found positive in 16% of transplant patients, thus indicating some possible association between RSV infection and lung transplant tolerance.

Real-time PCR based on RSV N gene has also been used to quantify RSV from the nasal aspirates of children [50]. The method was one log more sensitive than the conventional culture method. The real-time PCR assay resulted in 56% of positives, whereas the immunofluorescence assay had 41.3% and culture method had 45.3% of positives. Surprisingly, the RSV to GAPDH ratio did not differ in children with severe or nonsevere infection, raising confusion about the correlation of viral load to the severity of infection due to the intensity of viral replication, genetic susceptibility of the host and immune responses, thus, making it necessary to consider other factors besides the viral load.

A similar assay was developed for the detection of RSV using primer-probe sets from the RSV F gene [51]. A comparative analysis was made between the nested RT-PCR, real-time PCR, and ELISA, wherein real-time PCR was 25% more sensitive than the conventional nested PCR, thus making it more applicable for clinical samples. Additionally, the real-time PCR was performed in two steps to increase the sensitivity of the samples. This method was more rapid as the results were obtained in 3.5 to 4 hours upon receiving the samples. Several other reports also emphasize the applicability of real-time PCR in detection of RSV in immunocompromised patients [52]. Besides the RSV F and N gene, the RSV matrix gene and polymerase gene have also been used to detect RSV in children [53]. The latter method had the ability to quantify and classify RSV with high efficiency and rapidity. However, the sensitivity of the technique relies on the age of the patients.

#### 2.3.3. Multiplex PCR

The multiplex PCR approach has been used for the detection and subtyping of RSV and human influenza virus simultaneously [54] and as many as 18 respiratory viruses could be detected [55]. The method was based on the primer sets of hemagglutinin and nucleoprotein gene of influenza virus and nucleocapsid protein gene of RSV and found to be rapid, specific, and sensitive. However, the method suffered from the drawback that it could not distinguish between RSV A and B. This method could prove significant during a respiratory outbreak for surveillance. In such situations, multiplex real-time PCR assay, such as the commercial “Simplexa Flu A/B & RSV kit” that differentiates influenza A virus, influenza B virus, and RSV, is very useful [56]. Likewise, recently, a rapid and sensitive detection assay for RSV and other respiratory viruses was developed by Idaho Technologies christened as FilmArray (Idaho Technologies, Salt Lake City, UT). This is an automatic real-time molecular station with capability of nucleic acid extraction, initial reverse transcription, and multiplex PCR followed by singleplex second-stage PCR reactions for specific virus detection [57].

### 2.4. Microarray

Correlating gene expression signatures with disease progression of the patient's individual genetic profile is possible by comprehensive understanding and interpretation of the dynamics of biome-interactions in the lungs. This would result in more efficient therapy for respiratory diseases via the concept of personalized medicine, wherein the microarray finds its applications. Microarrays are the miniaturized assay platforms with a high-density array of immobilized DNA or protein. Hybridization of sample biomolecules to corresponding DNA or protein on the chip is detected, which allows determination of a variety of analytes present in the samples in a single experiment [60]. Microarray has proved to be a robust and reliable tool to understand the echelon of genomics, transcriptomics, and proteomics. Implementation of microarray with the metagenomic approach serves for rapid virus identification. Microarray based identification and characterization of viruses in clinical diagnostics are possible by designing oligonucleotide probes using the sequence data available in the public database. This approach is capable of discovering novel viruses, even though there are no conserved genes that can be targeted by sequencing. In the case of respiratory diseases, it can be executed by collecting the bronchoalveolar lavage enriched by nuclease treatment followed by filtration and extraction of total nucleic acids. Further, the nucleic acids are amplified by a random priming-based method, referred to as sequence independent single primer amplification, giving near-full-length reads of genomes of RNA or DNA viruses, which could then be compared to known viruses and used for designing the oligomers for the microarray [61]. Thus, sequencing coupled with microarray is a powerful high throughput diagnostic tool.

The utility of a metagenomics based strategy for broad-spectrum diagnostic assay using microarrays was demonstrated for screening viruses like Rhino virus, Parainfluenza virus, Sendai virus, Poliovirus, Adenovirus, and RSV from clinical samples [62]. A well-known example is “Virochip” which is a pan-viral microarray, designed to simultaneously detect all known viruses, and has comparable or superior sensitivity and specificity to conventional diagnostics [63, 64]. Similarly, an influenza microarray, “FluChip-55 microarray”, for the rapid identification of influenza A virus subtypes H1N1, H3N2, and H5N1 was developed [65]. The procedure is simple and follows few steps including RNA extraction from clinical samples, subsequently their reverse transcription, second-strand cDNA synthesis, and then PCR amplification of randomly primed cDNA. The hybridization of nucleic acids with probes is done by fluorescent dyes or by an alternative method of electrochemical detection. The latter method relies on a redox reaction to generate electrical current on the array for measurement. Incorporation of Cy3 fluorescent dye and hybridization to the microarray [66] has been empowered by specific algorithms to match the diagnostic needs, leading to the final and critical step of scanning and analysis [67].

The genes involved in the pathways of neuroactive ligand-receptor interaction, p53 signaling, ubiquitin mediated proteolysis, Jak-STAT signaling, cytokine-cytokine receptor interaction, hematopoietic cell lineage, cell cycle, apoptosis, and cancer were upregulated in RSV-infected BEAS-2B cells. RSV infection up-regulated 947 and 3047 genes at 4 h and 24 h, respectively, and 124 genes were common at both instances. Moreover, 1682 and 3771 genes were downregulated at 4 h and 24 h, respectively, and only 192 genes were same. Respiratory disease biomarkers like ARG2, SCNN1G, EPB41L4B, CSF1, PTEN, TUBB1, and ESR2 were also detected. RSV infection signs and symptoms render a partial consequence of host pathogen interaction, but the transcription profile is better exemplified by microarray. The transcription profiles of RSV infected mice lungs and lymph nodes showed gene expression of antigen processing and inflammation. The response is higher in lungs on the day 1 after RSV infection than day 3. The gene expression profile shortly after RSV infection can be accounted as a biomarker and can be scaled up for diagnostics with *in vitro* and *in vivo* profiles [68, 69].

Sometimes DNA microarrays do not confer their utility in very specific investigations in the case of personalized medicine wherein the manifestation of disease occurs at the transcription level. This situation may arise in complications of RSV with other associated disorders like asthma [70, 71]. As protein is the abundant functional biomolecule, reflecting the physiological or pathological state of the organ, protein microarray profile is an option to access under this situation [72]. Protein microarray has analytical and functional applications to study the protein-protein, protein-DNA, and protein-ligand interactions. These features enable the profiling of immune responses and are thus important for diagnostics and biomarker discovery. Protein microarray can serve as a rapid, sensitive, and simple tool for large-scale identification of viral-specific antibodies in sera [73, 74]. An extensive study was done during the 2002 SARS pandemic using coronavirus protein microarray to screen antibodies in human sera (>600 samples) with >90% accuracy and at least as sensitive as, and more specific than the available ELISA tests [73]. Thus, this system has enormous potential to be used as an epidemiological tool to screen viral infections.

### 2.5. Mass Spectroscopy

**Figure 3:**
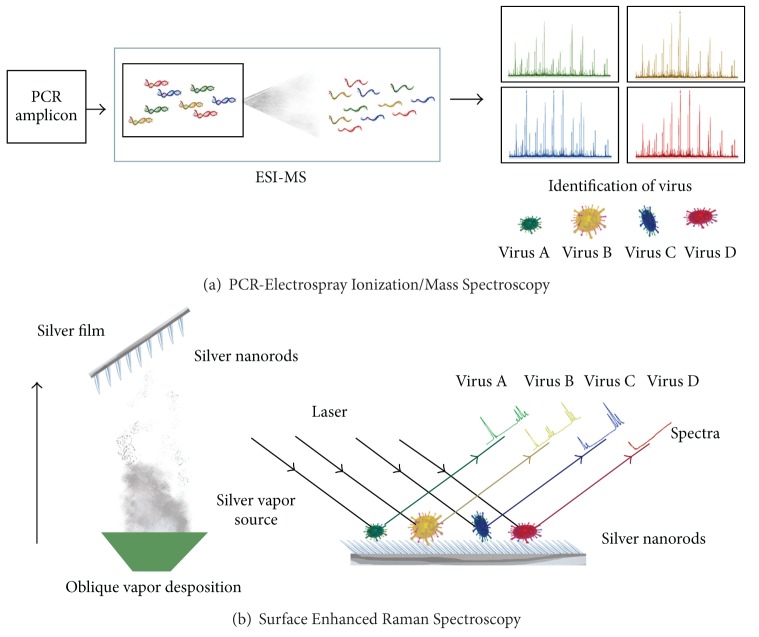
A schematic representation of biophysical method of RSV detection. (a) PCR-electrospray ionization mass spectroscopy and (b) Surface enhanced Raman spectroscopy.

Mass spectroscopy (MS) has become a method of choice for molecular investigation of pathogens as the reliability is reinforced due the well-characterized sequence information of nucleic acids or proteins and even for intact viruses [86, 87]. But pragmatic usage of MS is possible when coupled with various chromatography and affinity-based techniques. The combination of affinity based viral detention and nucleic acid based MS serves as a solution for low detection limits. Affinity-based methods employing nanotechnology can be used to find traces of target pathogen to improve detection limits. PCR amplification of pathogen nucleic acid combined with MS can be used as substitutes [88–90]. MS has the advantage of rapid identification of multiple viruses at the same time and even identifies the protein modification status [86, 91]. Now, MS is no longer confined to proteomics based analysis, and MS based genomics have become common practice. There are several variations of MS, like Matrix-assisted laser desorption/ionization mass spectroscopy (MALDI-MS), Surface-enhanced laser desorption/ionization mass spectroscopy (SELDI-MS), Bioaerosol mass spectrometry (BAMS), Pyrolysis gas chromatography mass spectrometry (Py/GC/MS), Capillary electrophoresis-MS, and Liquid chromatography mass spectrometry (LC-MS) are the few examples that are used for the identification of pathogens [89]. But the MS technique that promises robust practical application for pathogen detection is the electrospray ionization mass spectrometry (ESI-MS) [92, 93] Figure 3(a).

A notable ESI-MS for global surveillance of influenza virus was accomplished by Sampath et al. [75] by coupling MS with reverse-transcription PCR (RT-PCR). The study correctly identified 92 mammalian and avian influenza isolates (which represented 30 different H and N types, including 29 avian H5N1 isolates). The analysis showed more than 97% sensitivity and specificity in the identification of 656 clinical human respiratory specimens collected over a seven-year period (1999–2006) at species and subtypes level. The surveillance of samples from 2005-2006 influenza virus incidence showed evidence of new genotypes of the H3N2 strains. The study also suggested approximately 1% mixed viral quasi-species in the 2005-2006 samples providing insight into viral evolution. This study led to a number of RT-PCR/ESI-MS based detection methods for RSV and related respiratory viruses like influenza A and B, parainfluenza types 1–4, *adenoviridae* types A–F, *coronaviridae*, human bocavirus, and human metapneumovirus screening [76]. These assays had 87.9% accuracy, compared to conventional clinical virology assays and pathogens undetected by traditional clinical virology methods could be successfully detected. The advantage of RT-PCR/ESI-MS platform is the ability to determine the quantity of pathogens, detailed pathogen characterization, and the detection of multiple pathogens with high sensitivity and efficiency.

### 2.6. Nanotechnology Based Detection

Recent advancements in nanotechnology have changed the perception and perspective of research. When scaled down to nanometers, the properties of matter change and nanotechnology exploits these new properties and harnesses them with the existing technologies to exceptional capabilities. Techniques discussed below are examples of the nanotechnology based detection approaches which utilize basic traditional but indispensable detection techniques.

#### 2.6.1. Nanoparticle Amplified Immuno-PCR

Immuno-PCR, a combination of ELISA and PCR, is used widely for the detection of various bacterial and viral antigens with lower titers as meagre as zepto moles [58]. Perez et al. [59] reported a modification of immuno-PCR using gold nanoparticles for RSV detection. Target extraction was enhanced by using magnetic microparticles (MMPs) functionalized with anti-RSV antibody to capture the antigen (RSV). The MMP-RSV complex is then countered with gold nanoparticles functionalized with both, palivizumab (Synagis), an anti-RSV F protein antibody, and DNA sequence partially hybridized with a tag DNA sequence (fAuNP). The MMP-RSV-fAuNP complex is then heated to release the partially hybridized tag DNA sequence, which is then quantified from the supernatant by real-time PCR. These modifications enabled detection of RSV even at 4.1 PFU/mL. This assay offers a 4000-fold improvement in the limit of detection over ELISA and a 4-fold improvement over detection when compared with real-time RT-PCR [59].

#### 2.6.2. Live Cell RNA Imaging

Live cell imaging can provide the benefit of effective diagnosis and treatment, if the capability of identifying, monitoring, and quantifying biomolecules is developed. However, development of such systems for detection of viral agents is difficult, especially in the early stages of infection. With the advent of molecular beacon technology, it is now possible to track mRNA of host and RSV [32]. Based on this framework, a modification by oligonucleotide-functionalized gold nanoparticulate probe was suggested as an improvement, wherein a gold nanoparticle is functionalized with DNA hairpin structure by thiol linkage. The DNA is so designed that the loop portion has a complimentary sequence to RNA to be detected and the 5′ stem is linked to gold nanoparticle by thiol group and the 3′ end is linked to a fluorophore. On hybridization to the target RNA with loop, the fluorophore goes away from the quenching gold and the emission is tracked. Thus, live imaging of mRNA is possible. This mechanism of hairpin DNA functionalized gold nanoparticles (hAuNP) was executed by Jayagopal et al. in detecting RSV mRNA in HEp-2 cells [33]. This technique offers the advantage of real-time detection of multiple mRNA at the same time, including the mRNA of RSV and glyceraldehyde 3-phosphate dehydrogenase of the HEp-2 cell. Quantitative assay using this approach was possible using DNA hairpin structures functionalized to a gold filament, which is immersed in a capillary tube containing viral RNA and scanned for fluorescence. The set-up was able to detect as low as 11.9 PFUs, which was ~200 times better than a standard comparison ELISA.

#### 2.6.3. Quantum Dots

Immunofluorescence microscopy based detection of RSV, that is, direct fluorescent-antibody assay (DFA), is considered as gold standard [39], but the comparative efficacy of DFA with other assays does not seem to have reached a consensus [39, 40, 94–97]. The prime possible disadvantages and inconsistencies of DFA can be attributed to the fading of the dyes, conjugating antibodies with dyes, limited sensitivity due to background staining, and excitation at two different wavelengths [26–28]. To address these issues, fluorescent nanoparticles, that is, quantum dots (QDs), appear as promising candidates for field clinical diagnostics. Due to their inorganic nature, they are less susceptible to metabolic degradation. QDs are photostable; that is, they do not lose fluorescence on long exposure to light and can be excited at the same wavelength while radiating at different wavelengths and hence can be used for multiplexing [27]. Various successful attempts were made to ameliorate RSV detection using QDs. The progression of RSV infection in the HEp-2 cell line was studied using confocal microscopy by QDs probing F and G proteins and it was found that this method was more sensitive than real-time, quantitative RT-PCR, particularly at early infection [29]. This approach was used by Tripp et al. *in vitro*, on Vero cell lines and was extrapolated by an *in vivo* BALB/c mice study, which concluded the approach beyond diagnostics as it can be used for multiplexed virus and/or host cell antigen detection and intracellular tracking studies [28].

Flow cytometry is now widely used in diagnostics and the reliability is improved because millions of cells are analyzed at a time and comprehensive data is produced [30]. Tracking and targeting cellular proteins and the ease of multiple parameters correlation allow flow cytometry to be used for various qualitative and quantitative assays. Flow cytometer could detect RSV with sensitivity and reproducibility [31]. Agrawal et al. [26] showed that antibody conjugated QDs could detect RSV rapidly and sensitively using the principles of microcapillary flow cytometry (integrated with a fixed-point confocal microscope) and single-molecule detection. A 40-nm carboxylate-modified fluorescent nanoparticles and streptavidin-coated QDs were used in their study, which could estimate relative levels of surface protein expression.

#### 2.6.4. Gold Nanoparticle Facilitated Microarray

The FDA approved microarray systems, semiautomated respiratory virus nucleic acid test (VRNAT), and the fully automated respiratory virus nucleic acid test *SP* (RVNAT*SP*) (Nanosphere, Northbrook, IL) are examples of the microarray based detection systems for influenza A virus, influenza B virus, RSV A, and RSV B [56, 98]. These systems are based on the efficient detection of microarray based hybridization using gold nanoparticles. The hybridization between the oligonucleotide probes and target DNA/RNA is detected specifically by hybridizing them again to gold nanoparticles functionalized with oligonucleotide and the signal is generated by gold facilitated reduction of silver in the presence of a reducing agent [99].

#### 2.6.5. Surface Enhanced Raman Spectroscopy

Raman spectroscopy works on the basis of the inelastic scattering of monochromatic radiation like near infrared, visible, or near ultraviolet, interacting with an analyte with low-frequency vibrational and/or rotational energy. But the signal generated is low and hence the signal is enhanced using silver or gold matrix substrates. There are many modifications of Raman spectroscopy, but the most widely used one is the surface enhanced Raman spectroscopy (SERS) (Figure 3(b)) [77]. Direct intrinsic, indirect intrinsic and extrinsic detection are three major SERS detection configurations [78]. In contrast to IR spectroscopy, the Raman spectra can be obtained without the interference of water molecules and thus biological analytes can be studied in their native conformation. SERS is routinely used for various bioanalytical purposes due to the rapid and nondestructive detection of analytes with sensitivity, specificity, and precision even for a single molecule or live cells [79, 80]. SERS can be targeted to various analytes that may constitute DNA, RNA, proteins, or other organic compounds. Nucleic acids are the preferred candidates in biological SERS investigations, as the influence of base composition and sequence, conformation (local and/or global) of nucleic acids, or intermolecular dynamics with protein or ligand is correspondingly expressed as typical spectral signature [81]. Bacteria and viruses from various biological samples can be identified, characterized, and classified from clinical samples [77, 82]. SERS can distinguish between DNA or RNA viruses like Adenovirus, Rhinovirus, Rotavirus [83], and RSV [84]. Rotavirus is the common cause of gastroenteritis in children and the rapid and sensitive detection was demonstrated with SERS [83]. SERS is an established powerful tool for sensitive, expedited, and specific detection of various respiratory pathogens, like *Mycoplasma pneumoniae* and RSV. Using silver nanorod arrays (NA) platform for SERS, differentiation of *M. pneumoniae* in culture and in spiked and true clinical throat swab samples was achieved [82]. The notable sensitivity of SERS to resolve strain level differences for RSV strains A/Long, A2, ΔG, and B1 can be exploited for clinical diagnosis instrumentation [84].

## 3. Prevention

**Figure 4:**
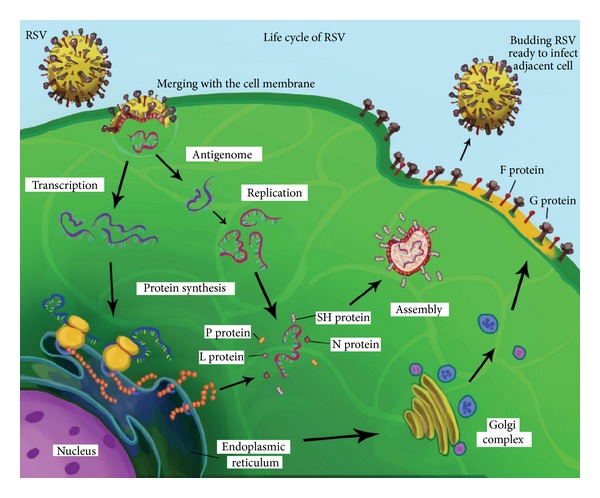
A schematic representation of RSV life cycle.

Prevention is the most important aspect of healthcare and has prime contribution to the culmination of the disease than the treatment measures. Effective preventive measures reduce the mortality and economic burden of the disease. Until now there is no effective vaccine for prevention for RSV. Direct or indirect contact with the nasopharyngeal secretions or droplets (sneezing, coughing and kissing), fomites, and food from infected patients can potentially transmit RSV. Live virus can survive on surfaces for several hours [100], but at the cellular level, the viral spread is a series of systematic events of invasion including viral attachment and fusion followed by viral replication and protein synthesis. The F protein accumulates in the host membrane and then surrounds the budding progeny viruses, thus spreading the infection to adjacent cells and exacerbating the infection (Figure 4). Thus these proteins are considered as the potential candidate for the development of prevention measures such as antibodies, DNA vaccines, and subunit vaccines.

### 3.1. Antibodies

**Figure 5:**
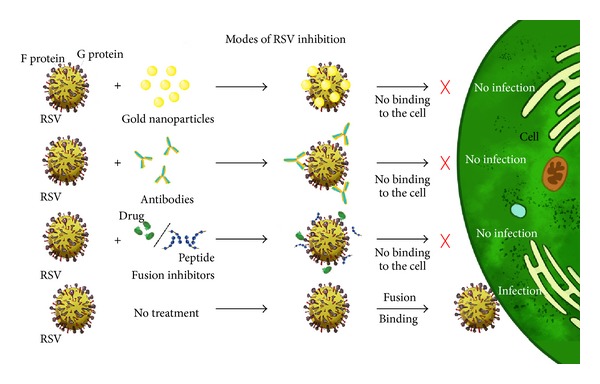
A schematic representation of various compounds inhibiting RSV binding to the cell.

RSV has three envelope proteins F, G, and SH. Both F and G are glycosylated and represent the targets of neutralizing antibodies. The RSV F protein emerged as a good vaccine candidate due to its conserved and vital role in cell attachment. Passive immunization is a direct approach to counter RSV (Figure 5). Initially, polyclonal antibodies from healthy human individuals resistant to RSV were successful in preventing RSV infection in high risk infants and these pooled and purified immunoglobins were popular as RespiGam. The monoclonal antibody specifically neutralizing F protein conferred effective protection against RSV as compared to RespiGam, and this licensed monoclonal antibody, palivizumab (Synagis), is now used to passively protect high risk infants from severe disease, thus replacing the RespiGam. The efficacy of the recombinant monoclonal antibody, palivizumab, has been tested for prophylaxis and therapy in immunocompromised cotton rats [101]. Repeated doses of palivizumab were required to prevent rebound RSV replication. Palivizumab is administered alone or in combination with aerosolized ribavirin. Palivizumab cannot cure or treat serious RSV disease but neutralization of RSV can help in preventing serious RSV infections. Motavizumab has been found to neutralize RSV by binding the RSV fusion protein F after attachment to the host, but before the viral transcription [102]. Viral entry was not inhibited by palivizumab or motavizumab when pretreated with RSV, but there was a reduction in viral transcription, thus inhibiting both cell-cell and virus-cell fusion most likely by preventing the conformational changes in the F protein needed for viral fusion.

The effective use of palivizumab is limited due to the cost and its use in infants with high risk of bronchiolitis based on the coverage by different healthcare systems [103]. In spite of these restrictions on palivizumab, it has a wide societal impact on use in infants with chronic lung disease due to premature birth or those with haemodynamically significant cardiac disease. According to the modified recommendations of the Committee on Infectious Diseases of the Centers for Disease Control and Prevention of RSV, palivizumab is recommended for infants with congenital heart disease (CHD), chronic lung disease (CLD), and birth before 32 weeks [104]. Minimum 5 doses are recommended irrespective of the month of the first dose for all geographical locations for infants with a gestational age of 32 weeks 0 days to 32 weeks 6 days without hemodynamically significant CHD or CLD. The new recommendations of the committee were aimed at the high risk groups including infants attending child care or one or more siblings or other children younger than 5 years living with the child. Also, the infants were qualified for receiving prophylaxis only until they reached 90 days of age. Palivizumab, although effective, is costly and thus is not beneficial to the recipients especially during the periods when RSV is not circulating. A cost effective means of producing RSV F neutralizing antibodies was experimented in phages and plants. Much success in this regard of palivizumab production was observed in the *Nicotiana benthamiana* plant system which offered glycosylation and high production at lower upstream and equivalent downstream cost, when compared to mammalian derived palivizumab. The efficacy of the plant derived palivizumab was more than the mammalian derived palivizumab or the plant derived human monoclonal antibodies in cotton rats [105].

### 3.2. DNA Vaccines

**Figure 6:**
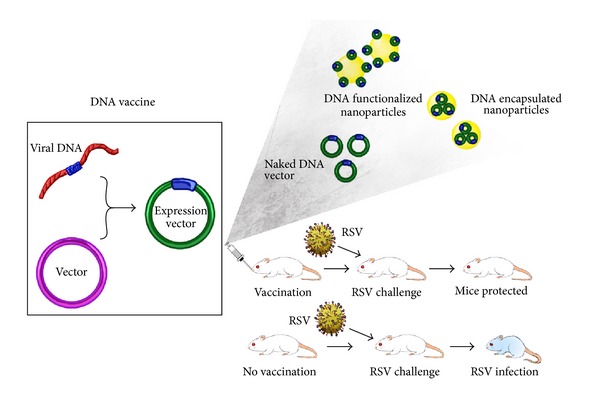
A schematic representation of a simple DNA vaccine administered as a naked DNA vector or functionalized nanoparticle or as an encapsulation for controlled delivery.

RSV genome codes structural and functional proteins that are immunogenic and referred for vaccine development against RSV. DNA based vaccines were developed based on these proteins because the concept was simple, as it involved a DNA fragment coding part or whole protein of RSV that was inserted into an appropriate expression plasmid vector under a constitutive promoter control (Figure 6). The initial work with this approach was successful in the expression in cells and *in vivo* murine models to eliminate the RSV infection, but the problem of RSV associated Th2 type immune response was persistent. This problem was attempted to be resolved by manipulating the parameters of choice of the protein to be expressed, the expression vector, adjuvants, formulations, and intracellular stability of the plasmid.

Mice challenged with the RSV-G construct had balanced systemic and pulmonary Th1/Th2 cytokines and RSV neutralizing antibody responses [106]. However, the RSV F protein gene is considered as a widely used and prospective target for the development of vaccines and is often a favourite model for DNA vaccines against RSV [107]. But the wild type RSV F protein expressed from DNA plasmid was poorly expressed, so a codon optimized DNA vaccine was designed for better *in vivo* expression and hence was more immunogenic and reduced the RSV titer [108]. To address the low immunogenicity of F protein, the antagonistic activity of RSV for IFN and immunopathology, a construct was designed by inserting the F gene into Newcastle disease virus (NDV) vector (NDV-F). This modification served the purpose of higher elicitation of IFN by NDV-F than RSV, protection against RSV infection without immunopathology and enhanced adaptive immunity in BALB/c mice [109]. Wu et al. [110] developed a DNA vaccination strategy against RSV using a mucosal adjuvant. Two DNA vaccine vectors, namely, DRF-412 and DRF-412-P containing residues 412–524 of the RSV F gene, were cloned into the phCMV1 DNA vaccine vector. The DNA vaccine vectors DRF-412 contained the cholera toxin gene region called ctxA2B acting as a mucosal adjuvant. The DNA vaccine was successfully expressed in mouse muscle tissue, which was confirmed by immunohistological analysis and RT-PCR. The immunized mice induced neutralization antibody, systemic Ab (IgG, IgG1, IgG2a, and IgG2b) responses, and mucosal antibody responses (Ig A) which mimicked the challenge with live RSV. The mice immunized with the DRF-412 vector contained less RSV RNA in lung tissue and induced a higher mixed Th1/Th2 cytokine immune response and had better protection than those immunized with the DRF-412-P vector, which was confirmed by lung immunohistology studies [110]. A rational approach to confer protection against various pathogens through single vaccination or more often known as combined or composite vaccines was employed for RSV, influenza A virus (INF-A), and herpes simplex virus type-1 [111]. Mice were immunized either by injection or by gene gun (gold beads as carrier of the plasmid DNA) with mixture of four plasmids: INF-A haemagglutinin (HA), INF-A nucleoprotein (NP), HSV-1 glycoprotein D (gD) and RSV glycoprotein F. This led to protection of mice from the respective pathogens of challenge; in addition, it also offered protection from *Mycoplasma pulmonis* challenge as well [111]. Recently several developments have been made in the field of recombinant vaccines. In one such approach, *Mycobacterium bovis* Bacillus Calmette-Gue'rin (BCG) vaccine was modified to carry RSV N or M2 and was tested and found to establish the Th1 type immunity in RSV challenged mice [112]. The recombinant vaccine also elicited the activation of RSV specific T cells producing IFN-*γ* and IL-2, along with reduction in weight loss, and lung viral protein load, thus establishing a Th1-polarized immune response.

Bactofection is bacteria mediated transfer of plasmid DNA into mammalian cells. Xie et al. employed this interesting way of delivering and expressing the DNA vaccine in mice against RSV [113]. This approach serves the purpose of naturally activating the immunostimulatory response of the host and also delivery of the DNA vaccine. Attenuated *Salmonella typhimurium* strain SL7207 expressing vector pcDNA3.1/F containing the RSV F gene was orally administered to BALB/c mice, triggering efficient antigen-specific humoral, cellular, and mucosal immunity [113, 114].

A novel method of developing RSV DNA vaccine was devised by replacing the structural genes with RSV genes in an attenuated strain of Venezuelan equine encephalitis virus (VEEV). VEEV has an ssRNA (+) genome and contains a strong subgenomic promoter. The replicon particles were prepared by providing helper RNAs encoding VEEV capsid and envelope glycoprotein which comprise structural proteins, and all of these, when transfected into Vero cells, resulted into a replicon particle that could independently synthesize the RSV protein, thereby activating the immune response and protection. The system could be modulated by the administration of helper RNAs. This strategy was applied to the mice and rhesus monkey models, conferring protection against RSV and a desirable extent of a balanced Th1/Th2 type immune response was received [115].

### 3.3. Subunit Vaccines

Several approaches have been considered for developing an effective vaccine against RSV including the formalin inactivated RSV vaccine developed in late 1960s, which instead resulted in an enhanced infection [116]. An efficient RSV vaccine would be the one with proper balance between immunogenicity and protection without any allergic response [8].

RSV F has widely been accepted as the vaccine candidate due to its conserved nature among various strains as well as among the other paramyxoviruses [8, 117–119]. An antigenic region corresponding to RSV F protein (region 255–278) was cloned into a vector with ctxA2 B gene of cholera toxin and named as rF255, which elicited a helper T cell type 1 immune response in mice. It also resulted in higher expression of serum neutralizing antibody in immunized mice [120]. Similarly, a multivalent recombinant protein was developed by cloning RSV F, M2, and G protein into a bacterial pET32a (+) vector (called rFM2G), which resulted in enhanced serum IgG titers [121]. rFM2G was also used in conjunction with flagellin as an adjuvant which did not increase the IgG titers. In an effort to address the issues of immunogenicity, several studies have used the approach of combining other viruses with RSV genes. One such study uses virus like particles (VLP) developed from NP and M proteins of Newcastle disease virus (NDV) and a chimeric protein consisting of cytoplasmic and transmembrane domains of NDV HN protein and ectodomain of the RSV G protein (H/G) [122]. These VLPs resulted in an immune response better than UV-inactivated RSV and provided complete protection in mice from RSV even at a single dose, with elicited neutralizing antibodies. The VLP-H/G-immunized mice did not show enhanced pathology as compared to FI-RSV. In another approach to solve the immunogenicity related problems in devising an RSV vaccine, a recombinant vector, based on either murine PIV type 1 or Sendai virus was used to deliver RSV G protein through reverse genetics [123]. This provided effective protection against RSV in cotton rats. Another similar study using Sendai virus (SeV) as a vaccine carrying RSV F gene provided protection against four pathogens including hPIV-1, mouse PIV-1, hPIV-3, and RSV [124]. This approach has been used to compare two vaccine models, one with SeV backbone and the other with PIV-3 backbone. The SeV based vaccine showed a decrease in RSV load in African green monkeys lung titers.

Another study with recombinant vaccine utilizes recombinant simian Varicella viruses (rSVVs) which express RSV G and M2 protein genes and was evaluated in the Vero cell line [125]. Such a recombinant vaccine approach could be very useful in elderly people with risk of infection with *Varicella* and RSV, as well as children with chickenpox and RSV. rSVV based vaccines resulted in enhanced immune responses to RSV antigens serving as suitable vaccines in rhesus monkeys. A very similar approach was adopted for developing a recombinant *alphavirus* or immune stimulating complex (ISCOM) antigen against RSV [126]. The recombinant vector was designed by using a self-abortive alphavirus called Semiliki forest virus carrying RSV F and G genes. The advantages of using such recombinant abortive viruses are that they can undergo viral infection just once, eliminating chances of adverse effects resulting from further infection, and infecting the host cell resulting in expression of the inserted RSV gene followed by humoral and cell mediated immune response. The ISCOM used was composed of *Quillaja* saponin, lipids, and RSV antigens, having adjuvant properties as well. ISCOM has been shown to enhance antibody production, T cell proliferation, and MHC class-I responses as well as RSV specific neutralizing antibodies, IgG and IgA. The recombinant vaccine SFV/F or SFV/FG resulted in IFN gamma response along with resistance to RSV infection without worsened RSV disease, whereas the results were opposite in ISCOM/FG with enhanced goblet cell hyperplasia post-RSV challenge.

### 3.4. Nanovaccines

Recently, several applications of nanotechnology have appeared in the development of vaccines popularly known as Nanovaccines. DNA vaccine is prone to rapid degradation when introduced into an animal system; so to increase the retention and increase the efficacy of the DNA vaccines, they can be encapsulated into a polymer that will protect and facilitate controlled release (Figure 5). Various synthetic or natural polymers are now experimented for targeted delivery and controlled release of the carrier. Chitosan is the polymer of great interest in respiratory disease treatment, because of its mucoadhesive property and biodegradability, which balances the purpose of longer retention and controlled release of carrier molecules encapsulated. Thus, chitosan nanoparticles are being developed against RSV. A DNA vaccine (DR-FM2G) constitutive expression vector consisting of antigenic regions of RSV F, M2, and G genes driven by human cytomegalovirus promoter was encapsulated using chitosan nanoparticles (DCNPs). The advantages of DCNPs are that it was more stable than naked DNA or chitosan and so ideal for protection against DNA degradation by nucleases. The sustainability of the DCNPs in mice was more than naked DNA which was correspondingly indicated by higher RSV protein expression evident by immunohistochemical and real-time PCR studies [127, 128]. A similar study was conducted by Eroglu et al. [129], where the highly conserved RSV F gene was cloned into pHCMV1 expression vector and encapsulated into poly-hydroxyethyl-methacrylate nanospheres coated by chitosan and transfected into Cos-7 cells. The transfection efficiency of the system was at par with commercially available transfecting agents. *In vivo* studies with the BALB/C mice also indicated F protein expression and reduced RSV infection.

A vaccine based on the recombinant RSV N gene rings, called N-SRS, enclosing a bacterial RNA has been developed and assessed intranasally in BALB/c mice [130]. N-SRS was adjuvanted with *E. coli* enterotoxin LT (R192G) with efficient protection against RSV and high titers of IgG1, IgG2a and IgA, and so forth. Although this nanovaccine elicited a mild inflammatory response in airways of the mice, it enhanced the expression of antigen specific CD4^+^ and CD8^+^ responses. In another novel approach, nanoemulsion (NE) was used as an adjuvant to mucosal RSV vaccine in a mouse model and found to provide significant protection against RSV, along with RSV specific humoral responses and an enhanced Th1/Th2 response [131]. The NE rendered the RSV ineffective when treated for 2 or 3 hours, with a reduction in viral titer up to 10-fold as compared to media controls. NE-RSV also led to higher expression of RSV specific antibody, with significant decrease in viral load, no hyperreactivity, and no Th2 cytokine induction. Thus, this novel approach seems to be a promising and safe vaccine option against RSV.

The bitter episode of formalin inactivated RSV vaccine has impeded the vaccine development and in fact has raised serious concern in the use of native RSV or its components. This approach was revived with a novelty, wherein the engineered RSV F protein aggregates formed nanoparticles and were used as vaccine, and these nanoparticles induced protective immunity in cotton rats [132]. To combat RSV, host neutralizing antibodies are always more preferred than the therapeutic antibodies. But the epitopes of the neutralizing antibodies are larger than those of the therapeutic neutralizing antibodies (palivizumab and motavizumab). Thus, the use of these epitopes for neutralizing antibodies as a vaccine requires the retention of immuno functional conformation while getting rid of the undesired protein. RSV F oligomeric protein nanoparticle was synthesized by inserting recombinant RSV F gene into Baculovirus and expressed in Sf9 insect cell lines. This resulted in high recombinant protein expression compared to native protein. This also resulted in the conformation of rosette nanoparticles which was the aggregate of multiple RSV F oligomers found to be immunogenic. The study was extrapolated to phase 1 human clinical trials, which showed its safety and efficacy against RSV [133].

### 3.5. Nutrition

Nutrition is not considered as treatment but is a prerequisite for homeostasis, and any nutritional imbalance attracts disorders and diseases. Supplementary nutrients could reverse the adverse effects of the disorders and diseases, so *in sensu* nutrition can serve both as treatment and prevention. Carbohydrate rich diets and diet lacking antioxidants like fruits and vegetables during pregnancy are suspected for RSV susceptibility [134]. Resveratrol (trans-3,4,5-trihydroxystilbene), a polyphenol from grapes has demonstrated reduction in RSV replication and inflammation [135, 136]. A study demonstrated that the cord blood deficient in vitamin D was associated with RSV bronchiolitis; the neonates were at higher risk of RSV in the first year of life [137]. Vitamin D inhibits NF-*κ*B signalling which is responsible for RSV inflammation without affecting the antiviral activity of the host [138]. Lower levels of micronutrients like zinc, copper, selenium and retinol (Vitamin A), and alpha-tocopherol (Vitamin E) were observed in the children affected by RSV and human metapneumovirus [139]. In one study, RSV infected children were administered vitamin A to compensate the lower vitamin A serum level which was found beneficial for children with severe RSV infection [140]. Also, probiotic diet has shown to boost resistance against pathogen by modulating immune response, as in case of RSV, *Lactobacillus rhamnosus* (a probiotic bacteria) treated BALB/c mice showed significantly reduced lung viral loads and pathology after the RSV challenge [141]. These studies indicate the importance and association of nutrition with RSV susceptibility.

## 4. Treatment

There are very limited treatment options available for RSV. However, there are many drugs for the symptoms associated with RSV infection. The target genes and proteins vital for RSV infection (discussed in Section 3) are important for developing preventative and treatment measures. The mode of action and potency of a drug determines the approach of prophylactic or curative application. Considering the proposed life cycle of RSV, theoretically, there are numerous modes to interfere with RSV infection, but these options may not be practical. Replication, transcription and fusion are the few target processes for drug development against RSV. A focus is therefore on development of potent drug which holds conformity in the human trials. Some of the approaches are described below that promise to be a potential treatment for RSV (Table 2).

### 4.1. Antiviral Drugs

Ribavirin or 1-[(2R, 3R, 4S, 5R)-3,4-dihydroxy-5-(hydroxymethyl)oxolan-2-yl]-1H-1,2,4-triazole-3-carboxamide is a widely used broad spectrum synthetic anti-viral drug for both DNA and RNA virus treatment. Oral and nasal administration of ribavirin for treatment of severe lower respiratory tract infections caused by RSV and influenza virus are an option [104]. Ribavirin is phosphorylated in the cells and competes with adenosine-5′-triphosphate and guanosine-5′-triphosphate for viral RNA-dependent RNA polymerases in RNA viruses. However, the mechanism of ribavirin differs for DNA viruses, as it is a competitive inhibitor of inosine monophosphate dehydrogenase (IMPDH), causing deletion of GTP and messenger RNA (mRNA) guanylyl transferase (mRNA capping enzyme) and adversely affecting protein synthesis [142, 143]. The exact mechanism of anti-viral activity of ribavirin against RNA and DNA viruses is still not clear. The usefulness of ribavirin against viruses is not only due to its anti-viral activity but also due to its capability to modulate the immune system. Ribavirin is suggested to have immuno-stimulatory effects on Th cells [144]. The derivatives of ribavirin such as viramidine, merimepodib, and other IMPDH inhibitory molecules like mycophenolate and mizoribine have shown antiviral activity against the hepatitis C virus, and hence, there is scope for investigating them as potential anti-RSV drugs [143, 145]. There are many other compounds that can inhibit RSV replication and a well-known compound RSV 604 ((S)-1-(2-fluorophenyl)-3-(2-oxo-5-phenyl-2,3-dihydro-1H-benzo[e][1,4]diazepin-3-yl)-urea) showed promising results against RSV [146].

A derivative of antibiotic geldanamycin 17-allylamino-17-demethoxygeldanamycin (17AAG) and 17-dimethylaminoethylamino-17-demethoxygeldanamycin (17DMAG) targeted against cancer has now attracted researchers due to its antiviral property. These compounds are HSP90 inhibitors and thus helpful against RSV, as RSV is dependent on heat shock protein (HSP90) for its replication [165]. The anti-RSV activity of these compounds was seen in human airway epithelial cells (HAEC) and is considered as drug resistant therapeutics, due to the highly conserved target chaperon protein. These compounds are also known to inhibit replication of HPIV, influenza virus, and rhinovirus. The anti-viral activity dose is not toxic to the cells and inhalation mode of treatment can increase the local efficacy and avoid unnecessary exposure to other organs [166].

### 4.2. Fusion Inhibitors

Recent advances in the development of anti-viral drugs include the fusion inhibitors. The fusion inhibitors are usually synthetic compounds or molecules interrupting the fusion of virus with the host cell usually by binding the fusion proteins (Figure 5). The fusion inhibitors have been widely studied as anti-viral agents in several viruses including HIV, RSV, Henipavirus, Hendra virus, Nipah virus, Paramyxovirus, metapneumoviruses, HIV, and RSV [11, 167–173]. The first reports of the use of peptide(s) as fusion inhibitors include the development of DP-178, a synthetic peptide based on the leucine zipper region of the HIV fusion glycoprotein gp41 [167], which showed an IC50 at 0.38 nM against HIV-1. Fusion inhibitors for the paramyxoviruses have also been developed based on the conserved region of the fusion protein F. The F protein is widely known for its conserved nature among the *Paramyxoviridae* family [7]. Lambert et al. [174] developed the fusion inhibitors belonging to the conserved heptad repeat (HR) domains of F1 region of F protein which is analogous to the peptides DP-107 and DP-178 of HIV gp41. These fusion inhibitors were tested against RSV, human parainfluenza virus 3, and measles virus, which showed antiviral activity specific to the species of origin. DP-178 is an FDA approved anti-HIV drug with International Nonproprietary Name (INN) Enfuvirtide and trade name Fuzeon. Out of the peptides tested, the peptide T-118 developed from RSV was the most effective, with an EC50 of 0.050 *μ*M. These fusion inhibitors were then further characterized and tested. It was shown that different fusion inhibitors derived from same HR region differ in their anti-viral activity [147]. The HR121 and HR212 peptides showed an IC50 of 3.3 and 7.95 *μ*M, respectively, against RSV. Similarly, another peptide inhibitor was developed from Rho-A which showed inhibition of syncytia formation induced by RSV [148]. RhoA (a small GTPase) is involved in many biological processes and was shown to bind the RSV-F protein at amino acids 146–155. A peptide derived from the RSV F binding domain of RhoA (RhoA77-95) was shown to inhibit RSV and PIV-3 infection and syncytium formation, block cell-to-cell fusion, and reduce viral titers and illness in mice.

The fusion inhibitors are not only limited to peptide inhibitors; but a range of chemical inhibitors have also been tested against RSV and benzimidazoles are well-known fusion inhibitors [175]. A lead compound was identified to subsequently synthesize an analogue JNJ2408068, a low molecular weight benzimidazole, which showed high anti-viral activity. It had an EC50 of 0.16 nM, 100,000 times better than that of ribavirin. This compound showed anti-fusion activity against RSV in a dual mode of action including prevention of cell-virus fusion activity as well as cell-cell fusion activity. However, it was found ineffective against other viruses of the family including HPIV-3 and measles virus. A vast screening of as many as 16,671 compounds (source ChemBioNet library) was conducted for anti-RSV activity *in vitro* and two novel compounds, N-(2-hydroxyethyl)-4-methoxy-N-methyl-3-(6-methyl[1,2, 4]triazolo[3,4-a]phthalazin-3-yl)benzenesulfonamide (named as P13) and the 1,4-bis(3-methyl-4-pyridinyl)-1,4-diazepane (named as C15) were mined, which reduced the virus infectivity with IC50 values of 0.11 and 0.13 *μ*M, respectively [176].

Recently, several groups have reported synthetic fusion inhibitors of RSV, especially the benzotriazole derivatives [177]. After evaluating the structure-activity relationship (SAR) of these compounds, named as series **1** compounds, it was observed that the topology of the side chains of these compounds is important and facilitates the modification of their physical properties, as many of these compounds showed poor therapeutic indices (cytotoxic effects) to the host cells tested. In order to address these issues, a second series of derivatives of the compound **1** were developed and evaluated for SAR and their functionality as fusion inhibitors [178]. These compounds were developed from **1**, which had a tolerant diethylaminoethyl side chain with both polar and nonpolar functionality against RSV and had a replacement of the benzotriazole with benzimidazole-2-one. These were potent inhibitors of RSV *in vitro*. These compounds were named **2** and had an additional structural vector absent in **1**, which accounted for enhanced potency as fusion inhibitors and served as the base for further development of fusion inhibitors. Further, these group **2** compounds were modified by introducing acidic and basic functional groups into the side chains [179]. The oxadiazolone had anti-RSV activity comparable to that of ribavirin, whereas the ester modified group **2** compounds were suitable for oral administration. These studies further led to the identification of a benzimidazole-2-one derivative called BMS-433771 which was an orally active RSV inhibitor [149]. Another compound studied was the 5-aminomethyl analogue **10aa** with potent anti-RSV activity towards BMS-433771 resistant RSV. The compound BMS-433771 was further modified at side chains and with the introduction of an aminomethyl substituent at the 5-position of the core benzimidazole moiety [180]. The aminomethyl substitution in the benzimidazole ring was found to enhance the antiviral activity.

Furthermore, the consecutive modification of benzimidazole resulted in benzimidazole-isatin oximes which were evaluated for anti-RSV activity [181]. The compound was analyzed for its antiviral activity, cell permeability, and metabolic stability in human live microsomes. Several other derivatives with modification such as O-alkylation and addition of nitrogen atoms to isatin phenyl ring were implemented also to enhance antiviral activity. Three compounds **18j**, **18i**, and **18n** were shown to have anti-viral activity against RSV in the BALB/c mice. Further, a compound RFI-641 was identified which was found to be the most potent anti-RSV agent inhibiting RSV both *in vitro* and *in vivo* and is in phase I clinical trials. RFI-641 is a biphenyl triazine synthesized by coupling diaminobiphenyl to two chlorotriazine molecules under microwave conditions [150]. RFI-641 was found to be effective against 6 laboratory and 18 clinical viruses at concentrations between 0.008 and 0.11 mM (0.013–0.18 mg/mL). The compound reduced the viral load to an extent of 1.7 logs in the African green monkey model and also in mice and cotton rats. In order to further enhance the antiviral activity of RFI-641, it was modified by replacing its triazine linkers with pyrimidine [182]. However, this modification did not have much difference in the anti-viral activity, thus rendering this modification not much of practical use. There are several novel nonbenzimidazole based compounds, showing anti-RSV activity *in vitro*, but a more polar compound thiazole-imidazole 13 was selected on the compound potency, moderate permeability, and low metabolic rate in rats, and more detailed *in vivo* studies are further anticipated [183].

### 4.3. Nanoparticles

It has been established that metals like silver [184] and gold [185] have anti-microbial activity, but cytotoxic effects of these reactive metals make them unsuitable for their use in humans. The reactivity and behaviour of metals can be modulated by reducing their size to nanoscale. Carbon nanotubes (CNTs) are emerging nanomaterials for biomedical application [186]. Polyvinylpyrrolidone (PVP) conjugated silver nanoparticles showed low toxicity to HEp-2 cells at low concentrations and exhibited 44% RSV inhibition [151] (Figure 6). Singh et al. used fusion inhibitor peptide functionalized gold nanoparticles and carboxylated gold nanoparticles of size 13 nm against RSV, which showed 83% and 88% inhibition of RSV, respectively [152]. Similar approach was employed by recombinant RSV F protein functionalized on gold nanorods [187]. The emergence of nanotechnology has opened new avenues for RSV treatment.

### 4.4. Antisense Treatment

RNA interference (RNAi) which is a normal cellular event has become a powerful means of controlling gene regulation. The interference mediated by siRNA was used against human immunodeficiency virus, poliovirus, hepatitis C, and parainfluenza virus (PIV) in cell culture [188–190]. The concept of inhibiting RSV infection using targeted antisense mechanism was applied by Jairath et al. by silencing the RSV-NS2 gene [191]. Following the RNAi approach, Bitko et al. designed siRNA against the P gene of RSV and PIV which protected mice against individual and mixed infections upon intranasal administration [192]. The effectiveness of siRNA action was observed with and without the use of transfection reagents. This approach was also effective when targeting the RSV-F gene [193]. Similar work on HEp-2 cell lines was replicated using four siRNA, designed to silence RSV F gene, which showed inhibitory action against RSV at various concentrations [194]. Silencing different RSV genes too had an inhibitory action on the RSV, a plasmid encoding siRNA which was complexed with chitosan targeting RSV-NS1 gene decreased RSV infection in BALB/c mice and Fischer 344 rats and also reduced the associated inflammation [195, 196]. Zhang et al. showed that siRNA nanoparticle targeting RSV NS1 gene resulted in increased IFN-*β* and IFN-inducible genes in A549 cells and in human dendritic cells, elevated type-1 IFN, and increased differentiation of CD4^+^ T cells to Th1 cells [196]. Also mice treated with siNS1 nanoparticles exhibited significant decrease in lung viral titers and inflammation.

An interferon-inducible enzyme, 2-5A-dependent RNase, present in higher vertebrates requires 5′-phosphorylated, 2′,5′-linked oligoadenylate (2-5A) for its endoribonuclease activity against single-stranded RNAs. This feature of 2-5A-dependent RNase is looked upon as an effective RSV treatment [197]. RSV upon infection elicits immune response of the host and particularly the interferon levels [68], and this phenomenon is exploited for the anti-RSV activity of 2-5A-dependent RNaseL. The endoribonuclease activity of 2-5A-dependent RNaseL was used for targeting RSV M2 gene specifically by covalent 2′–5′ oligoadenylate target antisense, which resulted in the reduction of RSV replication. The endoribonuclease activity had negligible effect with an inactive dimeric form of 2-5A linked to antisense, 2-5A linked to a randomized sequence of nucleotides, and antisense molecules lacking 2-5A and did not affect the other RSVs or cellular RNAs [198]. Additionally, to widen the range of the approach, the effects of modification of oligonucleotides and RNA target sites were studied [199]. This model was improved with respect to the specificity and activity by a chimera of 2-5A-antisense, christened as NIH351. The sequence information of RSV genome was used to develop NIH351 and was 50- to 90-fold more potent against RSV strain A2 than ribavirin [200]. Administration of siRNA in combination with ribavirin was recommended for effective treatment [201]. The parent molecule was chemically modified to further increase the *in vivo* stability and specificity and potency of NIH351. The resulting new version RBI034 was ~50% more effective than the parent molecule against RSV (strain A and B) and was not cytotoxic in the effective dose ranges. RBI034 treatment of African green monkeys shows promising results [202].

Alvarez and coworkers [153] came up with a new RSV-NS1 gene specific siRNA (ALN-RSV01) having a broad spectrum of antiviral activity that targeted the nucleocapsid gene of RSV. *In vivo* BALB/c murine studies demonstrated that intranasal dosing of ALN-RSV01 resulted in a 2.5- to 3.0-log-unit reduction in RSV lung concentration. To scale up this molecule for RSV treatment in humans, the safety, tolerability, and pharmacokinetics were tested on healthy adults, demonstrating its safety and tolerance in human subjects [154]. In the human clinical trials of ALN-RSV01, healthy subjects were grouped and administered either a placebo or ALN-RSV01 nasal spray for RSV. There was 44% reduction in the RSV infection in the subjects who received ALN-RSV01 without any adverse effect. Thus, this study in real terms has established a unique “proof-of-concept” for an RNAi therapeutic agent in RSV treatment [155]. ALN-RSV01 proved to be safe and was effective against RSV even in a complex clinical situation like lung transplants, which was a remarkable achievement [156]. It is the product of Alnylam Pharmaceuticals, Inc. and has completed phase IIb clinical trials.

Another approach of RNAi treatment to combat RSV is to decelerate the adverse effects of RSV mediated Th2 type immune response because the aggravated host immune response is more harmful than RSV infection itself. Particularly in neonates, RSV bronchiolitis increases IL-4*α* levels which results in increased Th2 response, so an antisense oligomer was synthesised for the local silencing of the IL-4*α* gene. Intranasal application of the antisense oligomers into a neonatal murine model reduced the Th2 type mediated pulmonary pathological signs of inflammation and lung dysfunction [203]. A combinatorial approach of the anti-sense oligomer against RSV and IL-4*α* would control RSV infection and the adverse effects of RSV mediated inflammation.

Phosphorodiamidate morpholino oligomers (PMOs) are the oligomers where the nucleobases are covalently attached with the morpholine ring replacing the deoxyribose sugar while the phosphodiester bond is replaced by the phosphorodiamidate linkage [157]. Morpholino chemical modification of RNA can be used as antisense with the advantages of specificity, *in vivo* stability, and targeted delivery. PMOs block the target complementary RNA and the target RNA fails to interact with the proteins and thus the RNA function is hindered. This is specifically true for mRNA and its translation. This phenomenon is different from antisense as the RNase H is not involved [204]. Hence PMOs can be designed containing the initiation codon against viruses to be anti-viral. Better intake into the cell and *in vivo* systems can be facilitated by conjugating cell penetrating peptide(s) (arginine-rich peptide (RXR) 4XB) to it. This approach was attempted against the RSV-L gene to inhibit RSV in cellular and murine models [205].

### 4.5. Ethnobotanicals

Various natural and synthetic chemical compounds have been screened for their application to treat RSV infections. Plants are rich sources of alkaloids, steroids, flavonoids, and other complex compounds that have medicinal value and medicinal plants contribute significantly to the traditional Indian and Chinese medicine. Ancient Chinese literature has descriptions of plant extract against respiratory diseases [206]. The exact active compound or action of the traditional formulations is not understood [207]. Now, modern assays have made it possible to get an insight into the mechanism and purification of active plant based compound. Extracts of *Lonicera deflexicalyx* (*Chin Jinyinhua*) were tested against RSV. The active compound from the extract was 3, 5-dicaffeoylquinic acid (CJ 4-16-4), which was isolated, and purified by a series of chromatographic processes. It is suggested that CJ 4-16-4 is a fusion inhibitor and the *in vitro* and *in vivo* studies suggest that it is a more effective RSV inhibitor than ribavirin [208]. Cytopathic effect (CPE) assay based screening showed that 27 of 44 herbs had moderate or potent anti-RSV activity [206]. The plant extracts from *Cinnamomum cassia*, *Cimicifuga foetida*, Sheng-Ma-Ge-Gen-Tang (SMGGT) (Shoma-kakkon-to), Xiao-Qing-Long-Tang (Sho-seiryu-to, so-cheong-ryong-tang), Ge-Gen-Tang, and Ginger (Zingiber officinale) show anti-RSV activity. These extracts probably inhibit RSV infection by blocking the F protein binding to the cell and some of the extracts even stimulate IFN-*β* production [158–163]. Some decoctions like modified Dingchuan (consists of *Salviae miltiorrhizae* radix, *Scutellariae radix*, *Farfarae flos* and *Ephedrae herba*), Liu-He-Tang (consist of 13 plant extract), and water extracts of Licorice (*Radix glycyrrhizae* and *Radix glycyrrhizae* Preparata) have shown effectiveness against RSV *in vitro*. Moreover, the modified Dingchuan decoction (MDD) exhibited anti-inflammatory and anti-viral effect in mice (SPF ICR mice) infected with RSV. MDD suppressed eotaxin, IL-4 and IFN-*γ* level in serum, and mRNA expression of TLR4 and NF-*κ*B in lungs of RSV infected mice [164].

## 5. Challenges in the Diagnosis, Prevention, and Treatment of RSV

Technology has provided enough capabilities for the detection of RSV in various sample types at various stages of infection using an array of techniques, but the challenge is, availability of these facilities at the correct time for a reasonable cost. The most important perspective of RSV diagnosis is the strategic management of choice between the point of care testing and central laboratory testing [36]. Though the point of care testing gives rapid detection advantage, it suffers from lower sensitivity and thus is a problem to be dealt with. Rapid diagnostic tools like RT-PCR/ESI-MS, microarray based semiautomated respiratory virus nucleic acid test (VRNAT) and the fully automated respiratory virus nucleic acid test SP (RVNATSP) (Nanosphere, Northbrook, IL) have proved their efficiency, but their application in routine clinical practice is still a challenge.

There are numerous molecules that can be potential antiviral drugs, but the screening of a vast number of compounds is cumbersome; hence, high throughput filtering is an essential part of drug development. As an example, when 313,816 compounds from the Molecular Libraries Small Molecule Repository were screened against RSV in HEp-2 cell line, only 409 compounds showed 50% inhibition of the cytopathic effects [209]. The challenge after this sophisticated screening is the translation into drugs by clearing the phases of *in vivo* animal studies and human trials. A setback for the development of therapies against RSV is the lack of a good animal model as they do not truly manifest effects of RSV infection as in humans. RSV experiments in various animal models like BALB/c mice, cotton rats, macaques, African green monkeys, owl monkeys, cebus monkeys, bonnet monkeys, olive baboons, and chimpanzees are evident in the literature. Small animal models like BALB/c mice and cotton rats are commonly used due to ease of handling and low cost, whereas the primate studies are conducted with more stringent regulations and bear heavy expenses [210, 211]. There are many aspects that need to be addressed in the challenges for vaccine development programs and the technological interventions to deal with RSV [212]. These challenges include safety issues concerning the subjects involved in clinical trials, as evident by the failure of formalin inactivated RSV vaccine and motavizumab at the clinical trial levels which resulted in undesired immunogenic responses in the patients involved [34, 116].

Though the present data conclude that RSV is one of the leading causes of morbidity and mortality in children and elders, there is no significant correlation between increased disease severity, respiratory deaths, and detection of any of the respiratory viruses [6, 213]. The situation is more intricate as some authors report that coinfection with non-RSV respiratory viruses tends to increase RSV severity [214] and also there is a hypothesis of a synergistic association of RSV with other viral or bacterial infections [215]. Thus, this topic is a subject of debate and these contradictions need clarification and consensus for proper treatment options. A link between atopy, asthma, and RSV was suspected for a very long time, but now there is supporting evidence for this possible relationship [216–218] and is also ascribed partly to genetic factors of RSV and the host [70, 71, 219, 220]. The activity of RSV in the community may be affected by many factors, including climate, air pollution [221], race/ethnicity [222], and social behavior of the population. Under these complex factors associated with RSV, it is established that early detection of risk factors and medical intervention can reduce the incidence of RSV [223]. A balance of execution and/or abeyance of prophylactic measures is critical with respect to the above discussed determinants [224]. There is limited data to correlate RSV global transmission dynamics with climate and population [2], due to which it is difficult to develop strategies for RSV prevention and treatment.

## 6. Conclusions

Currently, there is no vaccine or effective treatment against RSV, but the rapid and sensitive RSV detection is possible. The detection techniques are ameliorated by incorporating one or more methods and with the advancement in material science and biophysical capabilities, it has reinforced the development and design of RSV detection systems. However, an effective detection technique can be transformed into effective diagnosis by integrating it into the community health monitoring program at a reasonable cost. Prevention of RSV infection at present is limited to only high risk individuals with a limited efficacy. New preventive measures research like DNA vaccines, subunit vaccines, and nano-vaccines have reached animal trials. On the other hand, the RSV treatment approaches using antisense oligomers, fusion inhibitors, and benzimidazole drug have proceeded into clinical trials. The challenges associated with RSV management are categorically numerous. However, at the current pace of scientific research and development and with the implementation of scientific, commercial, and program recommendations to develop epidemiological strategies, it seems optimistic to have an effective diagnosis, prevention, and treatment solution for RSV in near future.

## Figures and Tables

**Table 1:** Comparison of RSV detection techniques.

Technique	Reference	Principle	Advantages	Drawbacks	Current usage status
(A) Fluorescence based methods					
(1) DFA	[24, 25]	Microscopic detection of RSV with specific antibody conjugated with fluorophore.	Easy procedure	Human error, fading of dyes	Research intent, Hospital based procedure, commercial diagnostic assays
(2) QDs	[26–31]	Detection of signals from fluorescent nanoparticles upon encounter with RSV either through microscopy or flow cytometry	Photostable, inorganic in nature, resistant to metabolic degradation	Toxicity, insolubility	Research intent
(3) Molecular beacon based imaging	[32, 33]	Hairpin DNA functionalized gold nanoparticle with fluorophore hybridization with target mRNA	Live cell imaging with real-time detection	Probable gene silencing, metabolic degradation	Research intent

(B) Immunoassays					
(1) ELISA	[34, 35]	Specific binding and colorimetric detection of antigen-antibody complex	Easy protocol, high specificity and sensitivity	Cumbersome, prone to human errors	Hospital based procedure, commercial diagnostic assays
(2) OIA	[36–38]	Presence of specific antigen-antibody complex formed alters the reflective surfaces properties which is visually detected	Easy, rapid, specificity, cost effective	Needs confirmation by other tests for negative samples	Research intent, not commercialized
(3) LFIA	[39–42]	Immuno-complexes detected chromatographically	Easy, rapid, handy, cost effective, FDA approved	Nonquantitative, limit of sample volume limits detection	Hospital based procedure, commercial diagnostic assays

(C) Molecular methods					
(1) LAMP	[41, 43–45]	Colorimetric/turbidimetric detection of isothermal amplification of DNA using specific primer	Sensitivity and specificity	Semiquantitative, designing compatible primer set	Research intent, not commercialized
(2) PCR	[46, 47]	Amplification of viral cDNA and visualization of PCR product	Rapid and sensitive than conventional culture methods	High limits of detection	Research intent, hospital based procedure
(3) Real-Time PCR	[48–53]	Real-time amplification of target DNA or cDNA	Rapid (3–5 hours), highly sensitive and very low limits of detection	Expensive	Research intent, hospital based procedure, commercial assay
(4) Multiplex PCR	[54–57]	Use of multiple primer and/or probe sets	Simultaneous detection of multiple pathogenic species or strains	Less sensitive	Research intent, hospital based procedure
(5) Immuno-PCR	[58, 59]	A combination of immunoassay and real-time PCR	Very low limits of detection, improved limits of detection over individual ELISA, and PCR (4000 and 4 fold. respectively)	Complex experimental design	Research intent, not commercialized
(6) Microarray	[60–74]	Hybridization of sample biomolecules to immobilized target DNA or protein on a chip	Highly sensitive, large scale identification of multiple pathogens; protein and nucleic acid targets	Cost-ineffective	Research intent, hospital based procedure, commercial assay

(D) Biophysical method					
(1) PCR-ESI-MS	[75, 76]	Mass spectroscopy of PCR-amplicons through electron spray dispersion	Highly sensitive and specific even at strain level and efficient multiple pathogens detection.	Expensive	Research intent, not commercialized
(2) SERS	[77–84]	Inelastic scattering of monochromatic radiation upon interaction with an analyte with low-frequency vibrational and/or rotational energy	Rapid and nondestructive detection of analytes with high sensitivity	Sample preparation	Research intent, not commercialized

**Table 2:** Comparison of different treatment approaches for RSV.

Treatment	Mechanism	Example	Remark	References
Antiviral drugs	Replication inhibition	RSV 604	Effective against RSV, but adverse effect on the host	[104] [142–146]
	Mutation	Ribavirin, viramidine, merimepodib		
	Inhibitor of inosine monophosphate dehydrogenase	Ribavirin, mycophenolate, mizoribine		
	Immunostimulatory effects	Ribavirin		
Fusion inhibitors	Inhibiting fusion protein attachment to cell	Peptide—HR121, HR212, RhoA Chemical—BMS-433771, RFI-641	Peptide fusion inhibitors promising anti RSV drug; chemical fusion inhibitors have side effects	[147–150]
Nanoparticles	Inhibiting attachment to cell	Silver nanoparticles, gold nanoparticles	Emerging field, conclusive studies required	[151, 152]
Antisense therapy	RNA interference	siRNA-ALN-RSV01 Phosphorodiamidate morpholino oligomers	Effective and safe; ALN-RSV01 completed phase IIb clinical trails	[153–157]
Ethnobotanicals	Probably fusion inhibitors, anti-inflammatory	Plant extracts—*Cinnamomum cassia*, *Cimicifuga foetida*, Sheng-Ma-Ge-Gen-Tang, Ginger, etc. Decoctions-modified Dingchuan, Liu-He-Tang, water extract of Licorice	Promising but conclusive studies required	[158–164]
